# Reducing Myosin II and ATP-Dependent Mechanical Activity Increases Order and Stability of Intracellular Organelles

**DOI:** 10.3390/ijms221910369

**Published:** 2021-09-26

**Authors:** Ishay Wohl, Eilon Sherman

**Affiliations:** Racah Institute of Physics, The Hebrew University, Jerusalem 9190401, Israel; ishaywohl@gmail.com

**Keywords:** Ca^++^, sub-diffusion, intracellular work, myosin II, blebbistatin, fractional Brownian motion (fBM), viscoelasticity, confinement, cell organization

## Abstract

Organization of intracellular content is affected by multiple simultaneous processes, including diffusion in a viscoelastic and structured environment, intracellular mechanical work and vibrations. The combined effects of these processes on intracellular organization are complex and remain poorly understood. Here, we studied the organization and dynamics of a free Ca^++^ probe as a small and mobile tracer in live T cells. Ca^++^, highlighted by Fluo-4, is localized in intracellular organelles. Inhibiting intracellular mechanical work by myosin II through blebbistatin treatment increased cellular dis-homogeneity of Ca^++^-rich features in length scale < 1.1 μm. We detected a similar effect in cells imaged by label-free bright-field (BF) microscopy, in mitochondria-highlighted cells and in ATP-depleted cells. Blebbistatin treatment also reduced the dynamics of the Ca^++^-rich features and generated prominent negative temporal correlations in their signals. Following Guggenberger et al. and numerical simulations, we suggest that diffusion in the viscoelastic and confined medium of intracellular organelles may promote spatial dis-homogeneity and stability of their content. This may be revealed only after inhibiting intracellular mechanical work and related cell vibrations. Our described mechanisms may allow the cell to control its organization via balancing its viscoelasticity and mechanical activity, with implications to cell physiology in health and disease.

## 1. Introduction

Intracellular content is viscoelastic [1,2] and organized [3]. It includes the actin cytoskeleton—an elastic polymer mesh that spans the cell volume and has a mesh size of ~50 nm [4,5]. This mesh is immersed in a viscoelastic and crowded medium with significant components that are gel-like and even in a solid phase [1,2,6,7]. 

Elasticity governs the mechanical properties of two main components of the cellular structure: the elastic cytoskeleton and the crowded viscoelastic intracellular medium [1,4]. Following that, elasticity is a dominant mechanical feature of living cells that enables them to maintain their shape and structure [4]. The cell elasticity also affects the diffusion of unbound molecules in the cytosol through hydrodynamic interactions between the different cell constituents [1]. 

The intracellular medium is organized in micro- and macro-compartments [3]. While the micro-compartments consist of large functional complexes of proteins that are not membrane-engulfed [7], the macro-compartments are typically engulfed with membranes and include structures such as the endoplasmic reticulum (ER), mitochondria, lysosomes, transport vesicles, secretory vesicles, etc. [3]. The abundance of such membrane-engulfed structures inside the cell creates multiple and complex diffusion barriers for molecules. In that way, a significant fraction of intracellular molecules experiences confinement as they impinge on intracellular membranes.

In a living cell, the combined effects of elasticity, viscosity, structural disorder, and mechanical work govern molecular diffusion [8,9]. Elasticity and structural disorder tend to hinder molecular motion, thus resulting in sub-diffusion, whereas mechanical work may result in extended molecular translocations and super diffusion [10]. Mechanical work inside living cells plays a significant role in cell physiology, utilizing molecular motors such as kinesin, dynein, and myosin II for direct and indirect transport of intracellular constituents [11]. Indirect motor activity includes the incoherent fraction of mechanical forces that are applied by molecular motors at multiple sites on the cytoskeleton. These incoherent forces impact the elastic cytoskeleton mesh [5], which in turn transfers those forces to the adjacent visco-elastic gel/solid macromolecular complexes. These complexes may further transfer those forces to unbound, yet hindered diffusing molecules in this crowded environment via hydrodynamic interactions [1]. Via that mechanism, intracellular mechanical work impacts important biophysical parameters such as intracellular diffusivity and homogeneity (or entropy) [12]. 

Several mathematical models are useful to describe aspects of molecular diffusion in the cell. Elasticity relates to fractional Brownian motion (fBM), which is one of the three main mathematical models that characterize intracellular random walks. A second main model is continuous time random walk (CTRW), which accounts for random walks with arbitrary distributions of translocations and waiting times. Molecular trapping and escape tend to generate such motion. Third, random walk on a fractal (RWF) accounts for molecular diffusion on a percolation structure, which has a non-Gaussian distribution of correlated translocations and relates to structural disorder and crowding [13]. fBM is a symmetric and a Gaussian diffusion process in which the increments are not independent and are correlated due to the elasticity effect. This stands in contrast to classical CTRW, for which the particles’ motion is not correlated. Notably, fBM and RWF are ergodic, while CTRW is not [9].

Diffusion is often characterized by its diffusion coefficient $k_{\alpha }\;$and the power of diffusion α, according to the mean-square displacement relation: $<r^{2}>\sim k_{\alpha }t^{\propto }$. These parameters are determined by the underlying mechanisms of diffusion. Elasticity tends to lower α in a negative correlation to the tension of the cytoskeletal mesh [8]. In contrast, intracellular mechanical work tends to increase diffusivity and α [8,12]. The contributions of elasticity, structural disorder, and mechanical work to α could be separated by inhibition of intracellular mechanical work. The summation of all of these contributions relates to the total measured α [12]. 

Recently, Guggenberger et al. [14] suggested theoretically that fBM in a one-dimensional finite interval may create inhomogeneity in the spatial distribution of the diffusing material inside that interval under steady-state conditions. The steady-state patterns of the spatial distribution of the diffusing particles inside that interval depend on the value of the Hurst index (H) of the fBM diffusing process. As shown by Guggenberger et al. [14], for fBM with H < 0.5, the diffusing particles tend to occupy the space near the interval borders less than at the center of the interval. The opposite occurs for H > 0.5, while for H = 0.5 (Brownian motion), the spatial distribution of the diffusing particles inside the interval is homogenous. 

Ions and relatively small molecules undergo diffusion in the cell cytosol and organelles and may serve as mobile tracers for studying diffusion in this complex medium. Ca^++^ is an important intracellular messenger and is responsible for multiple cell functions [15]. Ca^++^ is enriched in the endoplasmic reticulum (ER) [16] and mitochondria [17]. Fluo-4 AM (ester form) is an advanced fluorescence Ca^++^ indicator that enables the simple and sensitive imaging of intracellular and intra-organelle Ca^++^ levels in live cells [18]. Its molecular weight is 1097 g/mol and it can readily cross the cell or an organelle membrane via its ester moiety. Afterwards, intracellular esterases remove the ester moiety, and the Fluo-4 that now binds Ca^++^ can no longer cross membranes or exit the cell or the organelles [19]. As such, Ca^++^-Fluo-4 complexes may serve as an important example of intracellular molecules that organize inside multiple membrane-bound compartments and organelles.

Here, we aimed to study the dynamic organization of intracellular content in relation to intracellular organelles, especially in relation to the ER, which is the largest organelle and spans most of the cytoplasmic volume. For that, we studied the spatio-temporal organization of Ca^++^-Fluo-4 in Jurkat cells. We initially tested the effect of inhibition of intracellular mechanical work on the spatial and temporal distributions of imaged intracellular Ca^++^-Fluo-4 in living cells. In order to inhibit intracellular mechanical work, we utilized blebbistatin. This drug is an inhibitor of myosin II motors, which interact with the actin cytoskeletal network. We found that cell treatment with blebbistatin tends to reduce the Ca^++^-Fluo-4 homogeneity while reducing intracellular dynamics. We found similar effects in intracellular features, identified in label-free bright-field (BF) images of living cells before and after blebbistatin treatment, in mitochondria-highlighted cells and in ATP-depleted cells. Finally, we suggest a mechanism related to intracellular elasticity and fBM that could become detectable after blebbistatin treatment of the cells. Specifically, fBM may significantly affect the organization of material content in intracellular compartments, and thus determine overall intracellular homogeneity, and possibly the functional state of the cell and its physiology. 

## 2. Results

### 2.1. Myosin II Activity Increases the Homogeneity of Intracellular Ca^++^

In order to study the spatial and temporal distributions of intracellular Ca^++^ coupled to a fluorescence indicator, we imaged live Jurkat (CD4^+^) T cells—each cell before and after myosin II inhibition. For each cell, we obtained a series of 100 frames with a time interval of 0.3 s. To image intracellular Ca^++^, we used the Fluo-4 AM Ca^++^ fluorescence dye. To inhibit the mechanical activity of intracellular myosin II, we treated the cells with blebbistatin (see the Methods Section). Inhibition of myosin II activity is expected to reduce its mechanical interactions with the elastic actin cytoskeleton mesh [5]. In turn, this is expected to reduce the mechanical vibrations of that mesh and its adjacent viscoelastic medium, which both contribute a significant active component to intracellular diffusivity and motion [8]. 

**Figure 1:**
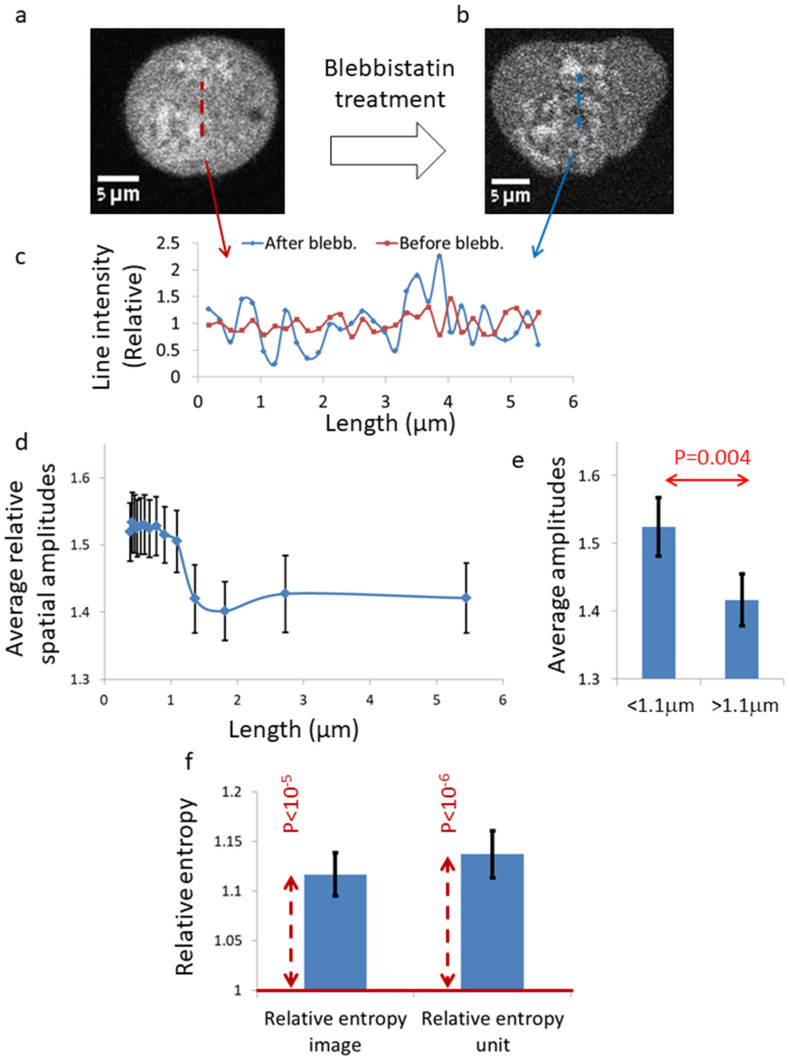
Spatial DFT analysis of Ca^++^-Fluo-4 in live T cells before and after blebbistatin treatment. (**a**) An image of Ca^++^-Fluo-4-highlighted, untreated T cell. (**b**) An image of the Ca^++^-Fluo-4-highlighted cell after 10 μM blebbistatin treatment. (**c**) Line scans of the relative light intensities [the pixels intensity divided by the average region of interest (ROI) intensity] in a line of 30 pixels, as illustrated in the corresponding image. (**d**) The average spectrum of the relative spatial amplitudes (i.e., the spatial amplitudes in a cell after blebbistatin treatment, divided by the corresponding amplitudes in the same cell before blebbistatin treatment) in all cells (N = 31). (**e**) The average relative amplitudes at spatial length scale < 1.1 μm in comparison to the average relative amplitudes at spatial length scale > 1.1 μm. (**f**) The average relative pixels’ information entropy (the pixels’ information entropy in a cell after blebbistatin treatment, divided by the pixels’ information entropy in the same cell before blebbistatin treatment) in all cells (N = 31). The pixels’ information entropy was calculated in two ways: first for all the ROI pixels’ values, and second by dividing the ROI to 36 units of 5 × 5 pixels and then averaging the pixels’ information entropy results of all units of a ROI. The red dotted arrows illustrate the difference in the average relative entropy values from a value of 1 (representing the null hypothesis). Error bars are SEM and *p*-values are shown in (**c**,**d**).

Ca^++^ highlighted by Fluo-4 (Ca^++^-Fluo-4) localized in intracellular organelles, such as mitochondria and ER [16,17] (Figure 1a). To confirm the relation of calcium repositories to the ER, we imaged live Jurkat cells that were stained with both Fluo-4 and ER tracker red (an ER fluorescent stain). This double staining confirmed that intracellular calcium repositories are related to the ER (see Supplementary Figure S3). Inhibiting intracellular mechanical work by myosin II through blebbistatin treatment increased cellular dis-homogeneity of the visible Ca^++^-rich features (Figure 1b and Supplementary Figure S1). We further studied the spatial distribution of intracellular Ca^++^-Fluo-4 using image spatial discrete Fourier transform (DFT) analysis for multiple (N = 31) cells before and after 10 μM of blebbistatin (see the Methods Section) [5]. We determined the relative DFT amplitudes as the DFT spatial amplitude in the cell after blebbistating treatment divided by the corresponding DFT spatial amplitude before blebbistatin treatment (see Supplementary Figure S2). These relative amplitudes are prominent in the spatial length of 0.39–1.09 μm (Figure 1d,e). Accordingly, inhibition of mechanical work by blebbistatin induces a significant increase in the intracellular heterogeneity at this length scale. This length scale is compatible with the typical size of intracellular organelles, such as mitochondria and ER segments. Thus, these results may indicate a change in the spatial distribution of Ca^++^-Fluo-4 in the ER or mitochondria. 

**Figure 2:**
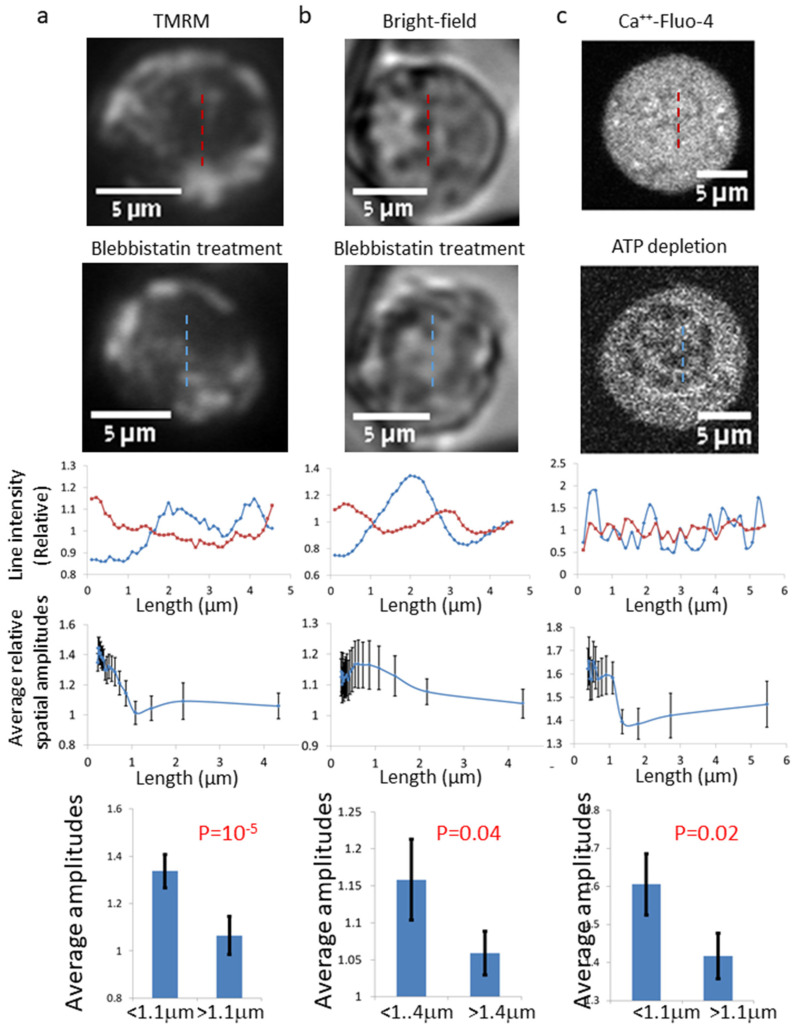
Inhomogeneity in living cells imaged by TMRM fluorescence (N = 21) (**a**), or label-free bright-field (BF) microscopy (N = 62) (**b**) and its relation to 10 μM blebbistatin treatment. Inhomogeneity in living cells imaged by Ca^++^-Fluo-4 fluorescence (N = 31) and its relation to ATP depletion treatment (**c**). From top to bottom rows: a representative cell before treatment, the same cell after treatment (blebbistatin or ATP depletion), its line scans of relative intensities (the pixels intensity divided by the average ROI intensity) before and after treatment, the average (for all cells) relative spatial amplitudes (see Figure 1) and the comparison of the average relative spatial amplitudes in two ranges of length. Error bars are SEM and *p*-values are shown.

We next aimed to see if the previously described spatial inhomogeneity of intra-organelle Ca^++^-Fluo-4 (mainly in the ER and mitochondria) could be extended to a more generalized intracellular inhomogeneity that could be explored through an additional intra-organelle probe or even in BF label-free images of cells. Tetramethylrhodamine methyl ester (TMRM) is known to concentrate inside active (ATP-producing) mitochondria. Accordingly, the spatial intensities’ fluctuations in TMRM-highlighted cells and in BF images were analyzed in a similar way to the analysis presented in Figure 1 (for Ca^++^-Fluo-4 cell images). Figure 2a,b further present the average results of the relative spatial amplitudes after spatial DFT analysis in cells before and after 10 μM blebbistatin treatment. 

Comparing the Ca^++^-Fluo-4 spatial distribution results in Figure 1 with the TMRM and BF label-free spatial distribution results in Figure 2 reveals similar patterns of results in both figures. The main elevated spatial amplitudes are at the same range between ~0.4 and 1.1–1.4 μm. This range is compatible with ER structures or mitochondrial length scale. Thus, the change in cell homogeneity after blebbistatin treatment could be demonstrated by analysis of both Ca^++^-Fluo-4, TMRM, and BF cell images. 

In order to confirm that the reaction of Ca^++^-Fluo-4-highlighted cells to blebbistatin was related to the reduction in intracellular mechanical work, we analyzed similar parameters in Ca^++^-Fluo-4-highlighted cells before and after ATP depletion. Intracellular ATP depletion was induced by 30 min incubation with 0.2 µM of the mitochondrial complex 1 inhibitor Rotenone, together with 10 mM of the glycolysis inhibitor 2-deoxy-D-glucose. Those results are presented in Figure 2c, and they show high similarity to the corresponding results in Figure 1. 

### 2.2. Myosin II Activity Increases the Mobility of Intracellular Fluo-4-Highlighted Ca^++^

**Figure 3:**
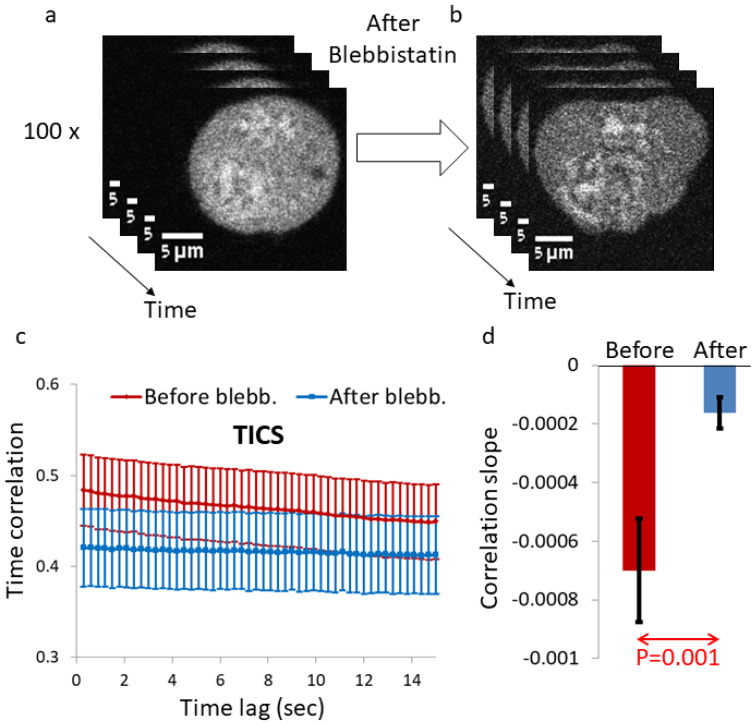
Temporal image correlation spectroscopy (TICS) in live cells highlighted with Ca^++^-Fluo-4 before and after blebbistatin treatment. (**a**) Multiple time-dependent images of a Ca^++^-Fluo-4-highlighted cell before blebbistatin treatment. (**b**) Multiple time-dependent images of a Ca^++^-Fluo-4-highlighted cell after 10 μM blebbistatin treatment. (**c**) The average TICS results for the cells (N = 31) before blebbistatin treatment and for the same cells after 10 μM blebbistatin treatment. (**d**) The average correlation slope in cells before blebbistatin treatment, in comparison to the average correlation slope in the same cells after blebbistatin treatment. Error bars are SEM and *p*-value is shown in (**d**).

Intracellular mechanical work increases both the diffusion coefficient and the power of diffusion [5,8,12]. The inhibition of that mechanical work is thus expected to reduce these parameters of diffusivity and intracellular dynamic. To verify that, we conducted temporal image correlation spectroscopy (TICS) analysis of the multiple frames of each Ca^++^-Fluo-4-highlighted cell (Figure 3a,b). The dynamics of those measured intra-organelle constituents were significantly reduced after inhibition of intracellular mechanical work by blebbistatin cell treatment (Figure 3c,d). Accordingly, it is reasonable to assume that both α and $k_{\alpha }$ are reduced under this condition [5,8,12].

**Figure 4:**
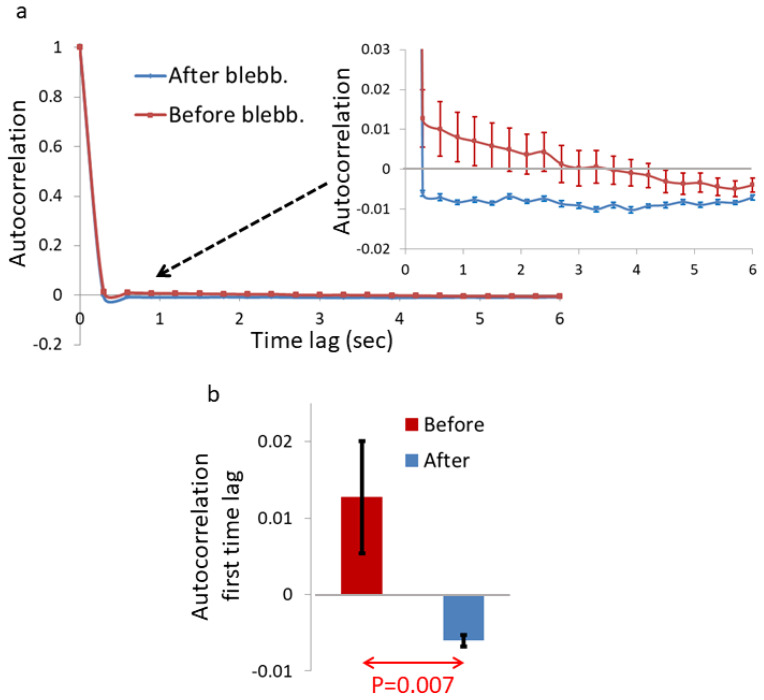
Autocorrelation of pixel intensities in the ROIs of Ca^++^-Fluo-4-highlighted live cells before and after blebbistatin treatment. (**a**) Average autocorrelation values of the pixel intensities in the ROIs in the cells (N = 31) before blebbistatin treatment, and of the same cells after 10 μM blebbistatin treatment. (**b**) Average autocorrelation results of the first time-lag in the cells before blebbistatin treatment in comparison to the corresponding results in the same cells after blebbistatin treatment. Error bars are SEM, and *p*-value is shown in (**b**).

Analyzing the autocorrelation of the time-dependent intensity of the pixels in the Ca^++^-Fluo-4 images reveals that the autocorrelation values drop rapidly to negative values in the cells after blebbistatin treatment, relative to cells before blebbistatin treatment (see Figure 4). 

We hypothesized that the reduced diffusivity of these intra-organelle constituents in cells after mechanical work inhibition may relate to the dominancy of elasticity and fBM. Under this condition, free Ca^++^-Fluo-4 molecules struggle to diffuse in the crowded and elastic gel/solid medium inside an organelle. Vibrations of that elastic medium (mostly thermal, after mechanical work inhibition) will create corresponding correlated motion of the embedded Ca^++^-Fluo-4 molecules. The complex vibrational motion of the elastic intra-organelle medium follows sub-diffusion fBM patterns with dominancy of negative correlations. This is supported by the intra-organelle medium being highly crowded [1], elastic [1,2], and therefore related to sub-diffusion fBM [2]. Those complex vibrations are reflected by the motion of multiple embedded Ca^++^-Fluo-4 molecules that follow the same patterns of negative correlations (Supplementary Figure S4). As can be seen in the schematic illustration in Supplementary Figure S4, the negatively correlated motion of multiple particles that are inspected by the ROI pixels will produce fluctuations of the intensity in those pixels that are also negatively correlated. According to that, the autocorrelation values of the time-dependent intensity fluctuations in these pixels will be negative and will relate to the negative correlations between the embedded particles’ motion. 

The complex and correlated vibrating motion of the Ca^++^-Fluo-4 molecules could be simulated by fractional Brownian noise (fBN). As mentioned above, the particles’ correlated vibrational motion is expected to be related to the pixels’ intensity autocorrelation results. Thus, simulated fBN under conditions that match motion in live cells (as detailed in the next section) should produce results that resemble the intensity autocorrelation results that are presented in Figure 4a. We conducted such a simulation for H = 0.3 and H = 0.6. Comparing the simulated fBN results (Supplementary Figure S5) with the intensity autocorrelation results (Figure 4a) indeed reveals the expected resemblance. Thus, the simulation results support the assumption that the negative intensity autocorrelation results under mechanical work inhibition reflect fBM dominancy of Ca^++^-Fluo-4 motion.

To conclude so far, the spatial inhomogeneity of intra-organelle content is increased after the reduction of intracellular mechanical work (Figure 1 and Figure 2). Under this condition, the dynamics of the intra-organelle content are also reduced (Figure 3). The dominant mechanism of motion under this condition is characterized by pronounced negative correlations (Figure 4). This indicates a more dominant role of cellular elasticity in molecular motion, now that active cellular fluctuations are reduced. Such motion may further relate to fBM.

It may further be expected that the inhomogeneity of intra-organelle content (or the spatial structures that are created inside those organelles) in low H fBM will be more stable as H becomes lower and the inhomogeneity effect becomes stronger. 

### 2.3. Live Cell Simulation Relates fBM to Spatial Distribution of Intra-Organelle Content

To further interpret the results of spatial and temporal fluctuations in intracellular Ca^++^-Fluo-4 levels in live cells and to interpret the stability of its apparent structures, we performed simulations with parameters that were adjusted to typical values in live cells.

Guo et al. [5] described active diffusion in live cells by monitoring multiple tracers, including nano-particles that were injected into the cells, intracellular vesicles, protein complexes, and specific proteins (Dendra 2–26 kDa fluorescence protein). They found that by monitoring relatively large time scales (>0.1 s), similar active diffusion patterns could be demonstrated for all these intracellular entities and tracers. In particular, intracellular vesicles and intracellular protein complexes showed similar diffusion patterns, including their α (Figures 4B and 6D in Guo et al.). In a previous work [12], we found *α* to be 0.79 in cells before ATP depletion and 0.70 in cells after ATP depletion. The corresponding H parameter values of fBM are 0.395 and 0.35. For the fBM simulation, we chose *K_α_* parameter values for protein complexes that were 0.0075 μm/s^α^ for normal cells and 0.001 μm/s^α^ for ATP-depleted cells (Figure 6D in Guo et al.). The borders of the fBM interval were considered to be reflective, and analysis of the particle probability of location inside the interval was conducted for 10,000 diffusion steps in order to reflect a steady-state condition. The results of that simulation for the length of the diffusing interval of 0.8 μm (which is compatible with the length scale of intracellular organelles, such as ER or mitochondria) and for different H values are presented in Supplementary Figure S6.

As can be seen in Supplementary Figure S6, the variance of probabilities of the fBM-diffusing particle along the limited interval is dependent on the H parameter values. Lower H = 0.3 values relate to ~3-fold larger probability of the particle to be in the center of the interval relative to localizing near the borders of the interval. For H = 0.5, the probability of the particle to be in the center is the same as the probability to be near the borders. Accordingly, the spatial distribution of particles under this Brownian motion condition is homogeneous. 

Following these results, it seems that in fBM sub-diffusion across finite intervals in living cells, lower H parameter values are related to significantly lower homogeneity of the intracellular content within those intervals. 

**Figure 5:**
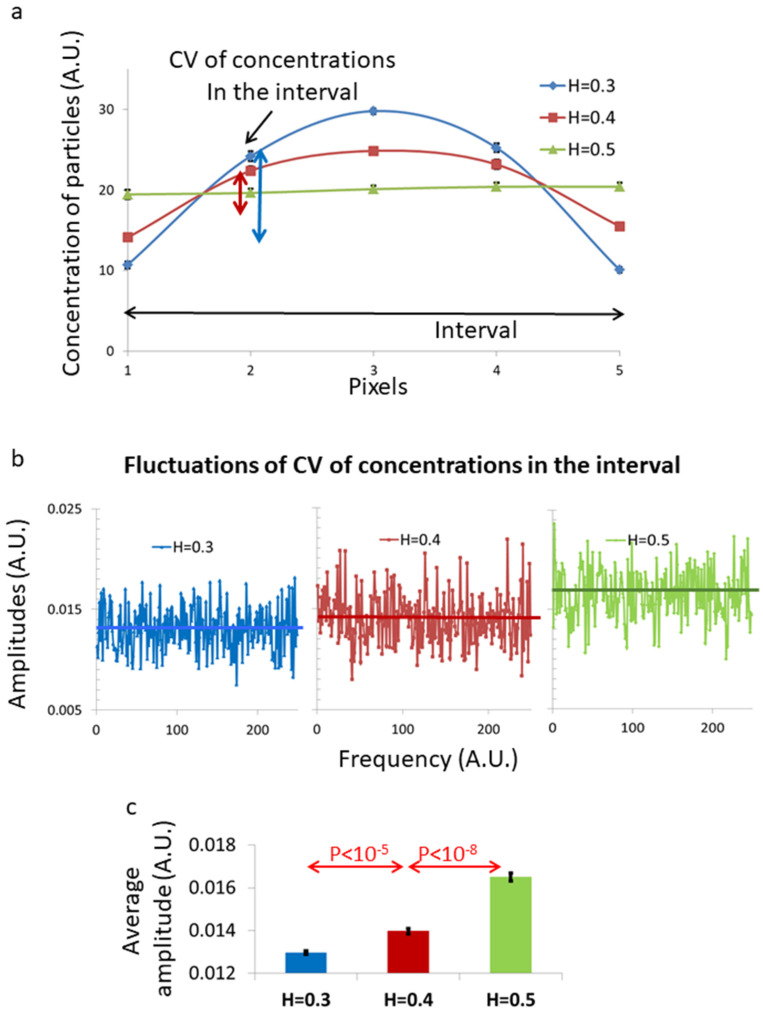
Simulation of coefficient of variation (CV) of concentration of particles and the fluctuations of those CV values across a 5-pixel interval under different fBM conditions. (**a**) Average simulated concentration of particles across the 5-pixel interval for different H values. Number of simulations was 10. Illustration of matched CV of concentrations for each spatial distribution of concentration across the interval due to the related H value is added. (**b**) The amplitude of fluctuations of those H-related CV values in 500 sequential simulations under each H condition. (**c**) The average amplitudes that were presented in (**b**) and their relation to H values. Error bars in (**a**,**c**) are SEM, and *p*-values are shown in (**c**).

Next, we analyze the fluctuations in the inhomogeneity that is created inside the interval by the fBM diffusion process. In other words, we analyze the stability or variation of the morphological structures that are created inside the interval. As can be seen in Figure 5a, the spatial distributions of multiple particles inside an interval depend on the standard deviation (SD) of the particles’ concentration along the length of the interval, relative to the average particle concentration or the total number of particles. The coefficient of variation (CV) describes that relation and defines the spatial inhomogeneity in the interval with no dependence on different average concentrations of particles inside the interval. The CV value of each fBM simulated process was calculated 500 times (each fBM process consists of 100 diffusion steps) and the fluctuation of CV values was analyzed. As can be seen in Figure 5b,c, the amplitude of fluctuations of the CV measure is dependent on the corresponding H values. As expected, low H values relate to lower amplitudes of fluctuations in the inhomogeneity inside the fBM interval. 

Accordingly, for low H fBM in a finite interval, the morphological structures that are created in that interval are more pronounced and stable. Thus, the H parameter, or the dominance of a sub-diffusive fBM process, controls each of these parameters: the extent of morphological structures that are created inside the diffusion interval and the stability of those morphological structures.

### 2.4. Intracellular Spatial Features Are Stabilized after Blebbistatin Treatment

**Figure 6:**
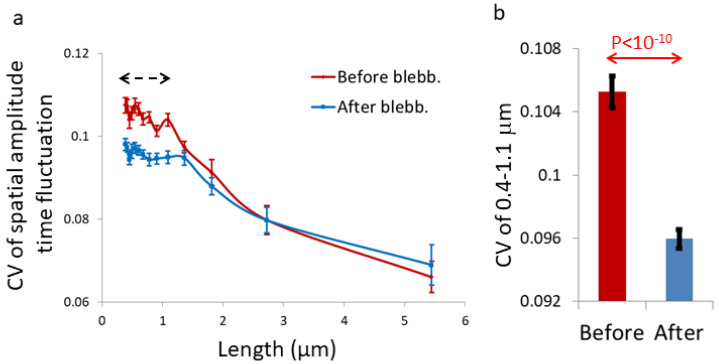
Temporal fluctuations (measured by the coefficient of variation (CV)) of the spatial amplitude in 31 live cells highlighted with Ca^++^-Fluo-4 before and after blebbistatin treatment. (**a**) Average CV values of the temporal fluctuations of the spatial intensity amplitudes in the cells before blebbistatin treatment, and of the same cells after 10 μM blebbistatin treatment. (**b**) Average CV values of the spatial amplitudes in the range of 0.39–1.09 μm in the cells before and after blebbistatin treatment. Error bars in (**a**,**b**) are SEM and *p*-value is shown in (**b**).

We further analyzed the time-dependent fluctuations of the spatial amplitudes shown in Figure 1. The extent of fluctuations of the spatial amplitudes describes the degree of stability of the morphological structures corresponding to these spatial amplitudes. As mentioned above, it is expected that in a sub-diffusion fBM process in a finite interval, the morphological structures and their stability will be negatively correlated to H values or be positively correlated to the dominance of the fBM process. As can be seen in Figure 6a,b, the degree of temporal fluctuations of the spatial structures at lengths below 1.1 μm is higher in cells before blebbistatin treatment relative to the same cells after blebbistatin treatment. 

In Section 2.1, we assumed that the dominant diffusion process in blebbistatin-treated cells inside the highlighted organelle intervals is fBM. This assumption was supported by the autocorrelation results in Figure 4. The presented results of temporal fluctuations of the spatial amplitudes are in accordance with this assumption. Thus, a dominant sub-diffusive fBM process inside those organelles in mechanical work-inhibited cells may also be related to the low temporal fluctuations of spatial amplitudes at the relevant length range (<1.1 μm). According to that, the spatial structures that are created under the dominant sub-diffusive fBM condition are not only more eminent but also more stable (less changing over time). 

## 3. Discussion

Here, we studied the effect of myosin II and viscoelastic diffusion (especially via fBM) on the organization of intracellular content. For that, we visualized intracellular Ca^++^-Fluo-4, TMRM, and label-free brightfield images in live Jurkat T cells before and after mechanical work inhibition by blebbistatin. Ca^++^, highlighted by Fluo-4, is localized in intracellular organelles such as ER and mitochondria, while TMRM is localized inside active mitochondria. The relation of Ca^++^ repositories to the ER was confirmed by double staining of live cells with Fluo-4 and ER tracker red (Supplementary Figure S3). We found that inhibiting intracellular mechanical work by myosin II through blebbistatin treatment increased cellular dis-homogeneity of Ca^++^-rich features in length scale < 1.1 μm. We detected a similar effect in TMRM and label-free BF cell images.

Notably, ER structural support and dynamic growth is mainly related to the microtubules network along with its molecular motors: kinesin 1 and dynein, and not to myosin 2 [20,21]. Accordingly, inhibiting myosin II by blebbistatin is not expected to impact the ER structure and dynamics. To confirm that, we first analyzed the spatial DFT amplitudes in the ER-stained cells and found no difference in those amplitudes before and after blebbistatin treatment (Supplementary Figure S3f). Next, we analyzed the TICS results in the ER-stained cells and we found that the slopes to the TICS decays were not changed in those cells after blebbistatin treatment (Supplementary Figure S3c). On the other hand, TICS results in those cells utilizing the Ca^++^-Fluo-4 staining revealed that the correlation decays are smaller in the blebbistatin-treated cells (Supplementary Figure S3b, in accordance with results in Figure 3). Following that, we conclude that although blebbistatin treatment reduces Ca^++^-Fluo-4 homogeneity and dynamics, it has no significant impact on the ER homogeneity and dynamics. Furthermore, blebbistatin did not change the size of the treated cells (Supplementary Figure S7). Mitochondrial structure may also be affected by myosin II activity that relates to the mitochondrial fission process [22]. Still, the time scale of that process is long (about 2 h) [23], such that it is not expected to significantly impact our results, which were obtained over much shorter time scales.

It is suggested that the morphological changes that we observed in the cells after blebbistatin treatment are related to the effect of blebbistatin because each of the cells was observed before and after blebbistatin treatment. In this way, the individual effect on each cell could be measured and the net effect of blebbistatin (as presented by the relative amplitudes or entropy) could be obtained. Although the cells were observed in a single cell resolution and the results were relative to the effect of blebbistatin, we cannot completely exclude other effects. To further relate the increase in cellular dis-homogeny to the effect of mechanical work inhibition by blebbistatin, we studied Ca^++^-Fluo-4-highlighted cells where their mechanical work was inhibited by ATP depletion. The obtained results (Figure 2c) were highly similar to the results obtained by blebbistatin (Figure 1). 

Blebbistatin treatment reduced the dynamics of the Ca^++^-rich features and generated prominent negative temporal correlations in their signals. We further wanted to account for the apparent changes in Ca^++^-highlighted complexes and their dynamics via consideration of possible diffusion processes in the cell. 

The intracellular medium is viscoelastic [1]. The cytoplasm is filled with large functional complexes of macromolecules [24]. Accordingly, as much as half of the cellular protein content may be in a solid phase [7]. Under this condition, the free volume for diffusion for other macromolecules that are not organized in large complexes is very small. As a result, their motion would displace the position of some of the organized macromolecules through hydrodynamic interactions [1]. That mechanism would account for the elasticity effect on the unbound moving molecule [24]. The diffusion coefficient of these mobile molecules under these conditions is expected to be hindered by as much as 10-fold, depending on the size of the molecule [25]. Even relatively small molecules such as the tracers of Ca^++^-Fluo-4 (with a size of ~1 KDa) or TMRM (with a size of ~0.5 KDa) used in this study would undergo viscoelastic diffusion.

As suggested by Guggenberger et al. [14], fBM dynamics inside a finite spatial segment (or interval) are expected to produce major inhomogeneity in the content of that interval. To complement our experimental findings, we conducted simulations of viscoelastic diffusion due to fBM in living cells. The simulations were conducted over a length scale of 0.8 μm, which is compatible with intracellular organelles such as ER segments or mitochondria. Under these conditions and for H = 0.3, the fBM process produced a three-fold concentration difference between the concentration of the interval content at the center of the interval relative to the concentration close to the borders. 

The dynamics of the cell content are influenced by three main factors: intracellular elasticity which relates to fBM, intracellular mechanical work, and intracellular structural disorder [13]. The first two factors could be more readily controlled by the cell as cross-linking and myosin II interactions determine the actin cytoskeleton elasticity [26], and since the activity of molecular motors (such as myosin II) determines intracellular mechanical work [5]. The dynamics of a particular constituent in a particular section of the cell are determined by the combined contributions of fBM controllable dynamics and mechanical work controllable dynamics. These contributions are on top of the contribution of diffusion within a percolation structure. The cell can readily control the extent of contributions from fBM and mechanical work, and the summation of these contributions relates to the total dynamics. As demonstrated in previous work [12], the power of diffusion α could be separated into two components of α: one that relates to mechanical work and the other that relates to other mechanisms that include the controllable elasticity. Inhibition of mechanical work reduces the total α, which under this condition reflects mainly elasticity and fBM. The α value under this sub-diffusion condition is less than 1 and H < 0.5. In this situation, this sub-diffusive fBM process is expected to induce a significant inhomogeneity in the distribution of the intracellular content due to multiple finite diffusion intervals that are created by the membranes of intracellular organelles. 

Following these considerations, we analyzed images of living cells, where their intracellular Ca^++^ repositories (mainly in the ER and mitochondria) were labeled with Flu-4 AM fluorescence dye. The Ca^++^-Fluo-4 complexes that are created inside the ER or mitochondria cannot cross the organelle membrane and exit the interval due to the removal of the ester moiety of the Fluo-4 by intracellular esterases. Hence, those complexes diffuse in finite intra-organelle intervals while embedded in a crowded viscoelastic gel/solid surrounding medium. Under this condition, the diffusion is inhibited and is most consistent with fBM [1]. 

Analyzing the dynamics of these intra-organelle Ca^++^-Fluo-4 complexes by TICS revealed that their dynamic is reduced after inhibition of intracellular mechanical work (Figure 3). We also found that these dynamics are characterized by strong negative autocorrelation values (Figure 4), which are expected in sub-diffusion fBM dynamics. These results support the assumption that blebbistatin treatment and mechanical work inhibition result in the dominancy of fBM as a mechanism of molecular sub-diffusion. Realizing that, the increase in spatial inhomogeneity after blebbistatin treatment in Figure 1 is assumed to be related to the increase in inhomogeneity that is caused by a sub-diffusion fBM process in a finite diffusion interval when H < 0.5. The expected stabilization of the dis-homogeneity morphological structures in a sub-diffusion fBM process in a finite interval that was demonstrated in simulations (Figure 5) was also demonstrated in live Ca^++^-Fluo-4-highlighted cells (Figure 6).

The results of double ER and Ca^++^ staining revealed that apart from the significant (and expected) spatial correlation between those highlighted features, the spatial cross-correlation between them decayed more steeply after blebbistatin treatment (Supplementary Figure S3d,e). These cross-correlation results suggest that blebbistatin affects the combined morphological organization of those two entities. Blebbistatin treatment only affects the morphological organization or dis-homogeneity of Ca^++^-Fluo-4 and not the ER (see Supplementary Figure S3f). Accordingly, it may be assumed that the change in Ca^++^-Fluo-4 homogeneity by blebbistatin interferes with the Ca^++^-Fluo-4 and ER cross-correlations. Ca^++^-Fluo-4 that becomes less homogeneous inside the ER lumen may explain the increased decay of the cross-correlation results that is dominant in length < 1.1 μm. 

The similar results that were obtained for TMRM and BF label-free images of cells before and after blebbistatin treatment suggest that this phenomenon that was initially demonstrated for intracellular Ca^++^ repositories may reflect additional and more general cellular consequences of this mechanism. The ER spans most of the cytoplasm volume. Thus, most of the cytoplasmic materials face a nearby limiting membrane, even if this material is outside the lumen of the ER. Accordingly, it seems that most of the cytoplasmic material, which is predominantly viscoelastic, may be significantly influenced by this mechanism of fBM-induced inhomogeneity. This may explain the similar and general results that we found by BF label-free cell imaging.

It seems that this mechanism that may broadly impact the cytoplasmic degree of inhomogeneity and entropy may also significantly affect the cell physiology. The addition of intracellular mechanical work increases α and counteracts the effect of fBM that induces inhomogeneity. In contrast, increasing the cytoskeleton tension (and elasticity) facilitates this effect that induces inhomogeneity and reduces entropy. In that state of reduced entropy due to sub-diffusive fBM, the creation of functional complexes and even new compounds may be enhanced. The creation of those complexes and new compounds should increase the cell organization and should considerably impact its physiological state. 

The cell can control the extent of mechanical work production and also the degree of its cytoskeleton elasticity by actin–myosin interactions and by controlling the degree of cross-linking [26]. In this way, it may readily control the resultant inhomogeneity and organization. As a considerable fraction of the cytoplasmic content is organized in functional multi-protein complexes, the degree of stability of these complexes is physiologically important. This stability would be directly influenced by the fBM process and facilitated by lower H values. On the other hand, too much inhomogeneity and stabilization will reduce the occurrence of dynamic interactions. In order for the cell to reach its preferable state of functional organization versus dynamics and flexibility, the degree of fBM-induced inhomogeneity or entropy reduction may be tuned. For instance, the amount of mechanical work in malignant cells is higher [5,27], while the intracellular tension [28,29] and dis-homogeneity [30] are lower. This may represent a special functional state of high dynamics, entropy and flexibility, along with their consequences. Thus, our suggested mechanism of fBM-induced inhomogeneity may be an important mechanism that affects normal and pathological cells’ physiology. 

To conclude, we showed a relation between inhibition of intracellular mechanical work to the organization of intracellular content with possible (patho) physiological implications.

## 4. Materials and Methods

### 4.1. Materials

Complete Medium (medium): RPMI-1640, heat-inactivated fetal calf serum (FCS), penicillin, streptomycin, glutamine, sodium pyruvate, and HEPES were from Biological Industries (Kibbutz Beit Haemek, Israel). Blebbistatin, tetramethylrhodamine methyl ester (TMRM), rotenone, and 2-Deoxy-D-Glucose were purchased from Sigma-Aldrich (St. Louis, MO, USA). Fluo-4 and ER tracker red were purchased from ThermoFisher scientific (Waltham, MA, USA). 

### 4.2. Cell Line 

Jurkat (human leukemic) E6.1 (CD4+) T cells were available to this study from previous research in the lab. Jurkat cells were maintained in RPMI-1640 medium supplemented with 10% FCS, 100 U/mL penicillin, 100 μg/mL streptomycin, 2% glutamine, 2% sodium pyruvate and 2% HEPES. Cells were maintained in a humidified atmosphere with a 5% CO_2_/air mixture at 37 °C. 

### 4.3. Sample Preparation 

For imaging, coverslip preparation was as follows: coverslips (#1.5 glass chambers, iBidi) were washed with acidic ethanol at room temperature (RT) for 10 min and dried at 37 °C for 1 h. Coverslips were then incubated at RT for 15 min with 0.01% poly-L-lysine (Sigma) diluted in water. This was followed by vacuum aspiration of the poly-L-lysine solution and drying of the coverslips at 37 °C for 1 h. Finally, cells were suspended in imaging buffer (composed of RPMI without phenol red + 10% serum + 25 mM HEPES) at a concentration of 1 million. Subsequently, 100,000–500,000 cells were applied onto the coverslips. 

### 4.4. Treatment of Jurkat Cells with Blebbistatin or Rotenone and 2-Deoxy-D-Glucose for ATP Depletion

Upon completion of measurements in all the cells and after recording the location of each cell, 10 µM of blebbistatin or Rotenone 0.2 mM and 10 mM of 2-deoxy-d-glucose were added to the cell medium. The samples were then incubated for 30 min on the microscope stage. At the end of incubation, each cell was measured again according to its recorded location.

### 4.5. Fluo-4, ER Tracker Red and Tetramethylrhodamine Methyl Ester (TMRM) Staining

Intracellular Ca^++^ was imaged using Fluo-4 fluorescence staining. From a working solution of 1 mM Fluo-4 in DMSO, 0.5 μL is added to 10^6^ Jurkat cells in Ca^++^-enriched imaging buffer solution and 15 μL of probenecid. Ca^++^-enriched imaging buffer solution has: 125 mM NaCl, 2 mM MgCl_2_, 4.5 mM KCl and 2 mM CaCl_2_. Cells were incubated for 1 h in the Fluo-4 solution and afterward, the cells were washed with PBS and then loaded on the coverslip in Ca^++^-enriched imaging buffer solution. Intracellular Ca^++^ and ER were both imaged using Fluo-4 and ER tracker red fluorescent staining. The Fluo-4 staining followed the previously described procedure. For ER staining, we added ER tracker red in a final concentration of 1 μM from a working solution of 1 mM in DMSO. The cells were then incubated for 30 min in PBS with calcium, magnesium and dextrose. Afterwards, the cells were washed with PBS and then loaded on the coverslip in Ca^++^-enriched imaging buffer solution. From a working solution of 100 μM in DMSO, TMRM was added to the cells’ imaging buffer medium at a final concentration of 50 nM. The cells were then incubated for 30 min and then washed with PBS and loaded on the coverslip in imaging buffer solution. 

### 4.6. Microscopy

Bright-field (BF) microscopy:

We conducted BF microscopy of live T cells using a motorized Olympus inverted IX81 microscope (Tokyo, Japan) with a halogen 12 V/100 W lamp light source. The microscope was equipped with a sub-micron Marzhauser-Wetzlar motorized stage type SCAN-IM, with an Lstep controller (Wetzlar-Steindorf, Germany). The condenser NA was 0.55 and the type of illumination was Koehler. A 60× air objective with NA = 0.70 was used for the BF images. Images were collected using a 12-bit cooled, highly sensitive ORCA II C4742-98 camera (Hamamatsu, Japan). Image acquisition time was 1 ms, in 16-bit TIF format, each having 1344 × 1024 pixels, and a physical dimension for each pixel of 110 nm at 60× magnification on the microscope working plane. Image stacks were generated by taking 200 serial images with an acquisition time of 0.5 s per frame. Image binning of 10 sequential gray values was used for the spatial entropy analysis. 

Confocal microscopy:

We conducted confocal microscopy of live T cells that were stained with the Fluo-4 fluorescent Ca^++^ indicator. Cells were scanned using the FV-1200 confocal microscope (Olympus, Japan) equipped with an environmental incubator (temperature and CO_2_). An image scan was performed using a 60×/1.42 NA oil objective, and for the Flou-4 imaging, excitation was at 488 nm and emission at 505–550 nm. Stacks of images were generated by taking 100 serial images with an acquisition time of 0.3 s for each frame of 140 × 140 pixels (with a 176 nm pixel size).

### 4.7. Data Analysis

ROIs of 30 × 30 pixels for the confocal microscopy experiment and 40 × 40 pixels for the BF microscopy experiment were determined at the center of each analyzed cell. The ROI size and its location were kept unchanged during the repeated acquisitions. The analyses that are described in the sections below were conducted utilizing those ROIs.

### 4.8. Image Correlation Spectroscopy (ICS) Calculations

The temporal correlations (TICS) and cross-correlations were calculated for each time-dependent region of interest (ROI) according to a generalized spatiotemporal correlation function [31]:

$$\delta i\left(x,y,t\right)=i\left(x,y,t\right)-{<i\left(x,y,t\right)>}_{t}$$

$$r\left(\xi ,\eta ,\tau \right)=\frac{<\delta i\left(x,y,t\right)\delta i\left(x+\xi ,y+\eta ,t+\tau \right)>}{{<i\left(x,y,t\right)>}_{t}{<i\left(x,y,t+\tau \right)>}_{t+\tau }}$$

where x,  y,  and t are the spatial and time coordinates, ξ,  η,  and τ are the spatial (horizontal and vertical) and temporal lags respectively, and i  stands for light intensity. All TICS correlation values were normalized using the corresponding zero correlations. For each stack of 100 images, a ROI of 30 × 30 pixels was defined at the center of each cell. Then, the normalized temporal correlation values were defined for each ROI in each cell and condition.

### 4.9. Image Spatial Discrete Fourier Transform (DFT) Analysis

Image spatial discrete Fourier transform (DFT) analysis was conducted for each ROI in each of the 100 time-dependent images. In each dimension, the sequence of pixels intensities along the ROI was analyzed by DFT and the average results of the vertical and horizontal pixels’ sequences of each time-dependent ROI were further averaged to obtain the final spatial DFT of that cell and condition. 

### 4.10. Information Entropy Calculations

For each stack of images, the ROI at the center of each cell was used for the information entropy calculations. The information entropy was calculated for all of the ROI pixels at once or for 36 units of 5 × 5 pixels, which the ROI was divided into, and that were then averaged. Information entropy values were calculated as follows: $E=-\sum P_{i}Ln\left(P_{i}\right)$, where $P_{i}$ is the probability to obtain intensity *i* and summation is overall intensity values. 

DFT and correlation analyses were carried out using Matlab R2017b (MathWorks, Natick, MA, USA). 

### 4.11. Simulation of fBM

For the simulation of fBM, we used the Matlab script for: Fast (exact) fractional Gaussian noise and Brownian motion generator by B. Scott Jackson (2005).

### 4.12. Statistical Analyses

The acquired data were exported to Excel spreadsheets (Microsoft Office Professional plus 2010, Microsoft Inc., Redmond, WA, USA) for graph presentation and for statistical analysis with the Real Statistic Resource pack. Significance of differences between groups was calculated using the analysis of variance (ANOVA) single-factor function or *t*-test for paired samples, with statistical significance set at *p* < 0.05. The magnitude of deviations from the mean are represented as standard errors of the mean (SEM): $SEM=\frac{SD}{\sqrt{N}}$.

## Data Availability

The authors declare that all data that supports the findings of this study are available within the article and its Supplementary Information and from the corresponding author on reasonable request.

## Supplementary Materials

The following are available online at https://www.mdpi.com/article/10.3390/ijms221910369/s1.
