# Supplementary material for: Reducing Myosin II and ATP-Dependent Mechanical Activity Increases Order and Stability of Intracellular Organelles
Source: Int J Mol Sci. 2021 Sep 26;22(19):10369. doi: 10.3390/ijms221910369 (PMC8508806; doi:10.3390/ijms221910369)
Supplement: Supplementary file 1 [file ijms-22-10369-s001.zip › ijms-1362359-supplementary.pdf]

International Journal of *Molecular Sciences*

MDPI

Article

Reducing Myosin II and ATP-Dependent Mechanical Activity Increases Order and Stability of Intracellular Organelles

Ishay Wohl and Eilon Sherman *

Racah Institute of Physics, The Hebrew University, Jerusalem 91904, Israel; ishaywohl@gmail.com
* Correspondence: eilon.sherman@mail.huji.ac.il

Citation: Wohl, I.; Sherman, E.; Reducing Myosin II and ATP-Dependent Mechanical Activity Increases Order and Stability of Intracellular Organelles. *Int. J. Mol. Sci.* **2021**, *22*, 10369. https://doi.org/10.3390/ijms221910369

Academic Editor: Herbert Schneckenburger

Received: 15 August 2021
Accepted: 23 September 2021
Published: 26 September 2021

Publisher's Note: MDPI stays neutral with regard to jurisdictional claims in published maps and institutional affiliations.

Copyright: © 2021 by the authors. Submitted for possible open access publication under the terms and conditions of the Creative Commons Attribution (CC BY) license (https://creativecommons.org/licenses/by/4.0/).

1. Supplementary Data

1.1 Relative Intensities Line Scans in 4 Cells before and after Blebbistatin Treatment

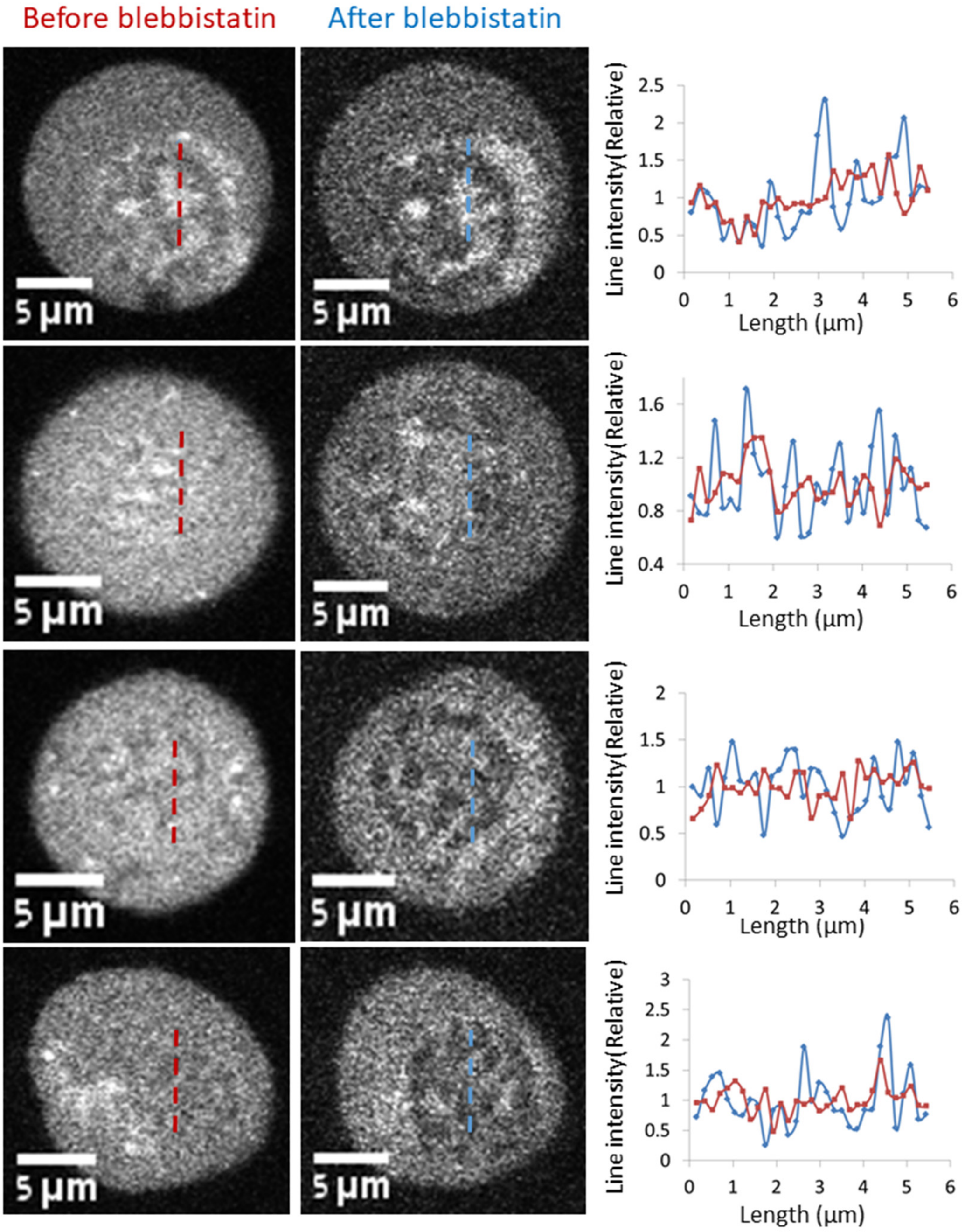

Figure S1. Ca^{++}-Fluo-4 in 4 live T cells before and after blebbistatin treatment. From left to right: 4 representative images of Ca^{++}-Fluo-4 highlighted, untreated T cell; the 4 correspondent images of the same cells after 10 µM blebbistatin treatment; the corresponding line scans of the relative light intensities (the pixels intensify divided by the average ROI intensity) in a line of 30 pixels as illustrated in each of the relevant image.

1.2 The DFT Spatial Amplitudes of the Cells before and after Blebbistatin Treatment

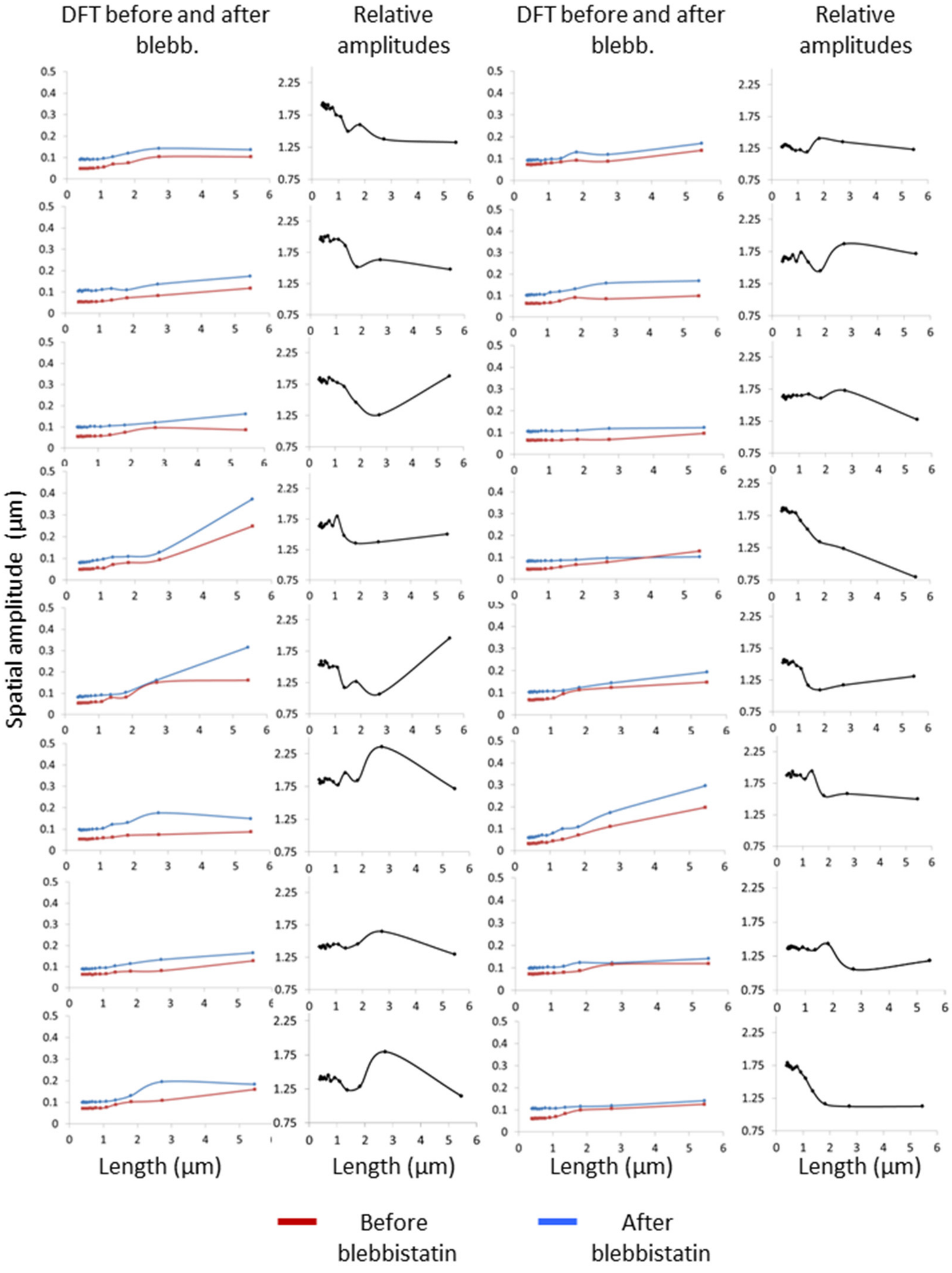

Figure S2. The spatial DFT amplitudes of light intensity spatial fluctuations in Ca^{++}-Fluo-4 highlighted live T cells. DFT results were measured in each cell before and after 10 µM blebbistatin treatment (red and blue curves, respectively). The corresponding relative amplitudes (the DFT amplitude after blebbistatin divided by the corresponding DVT amplitude before blebbistatin) are presented on the right side of each graph (black curves).

1.3 Combined Imaging of ER and Ca⁺⁺ in Live Cells before and after Blebbistatin Treatment

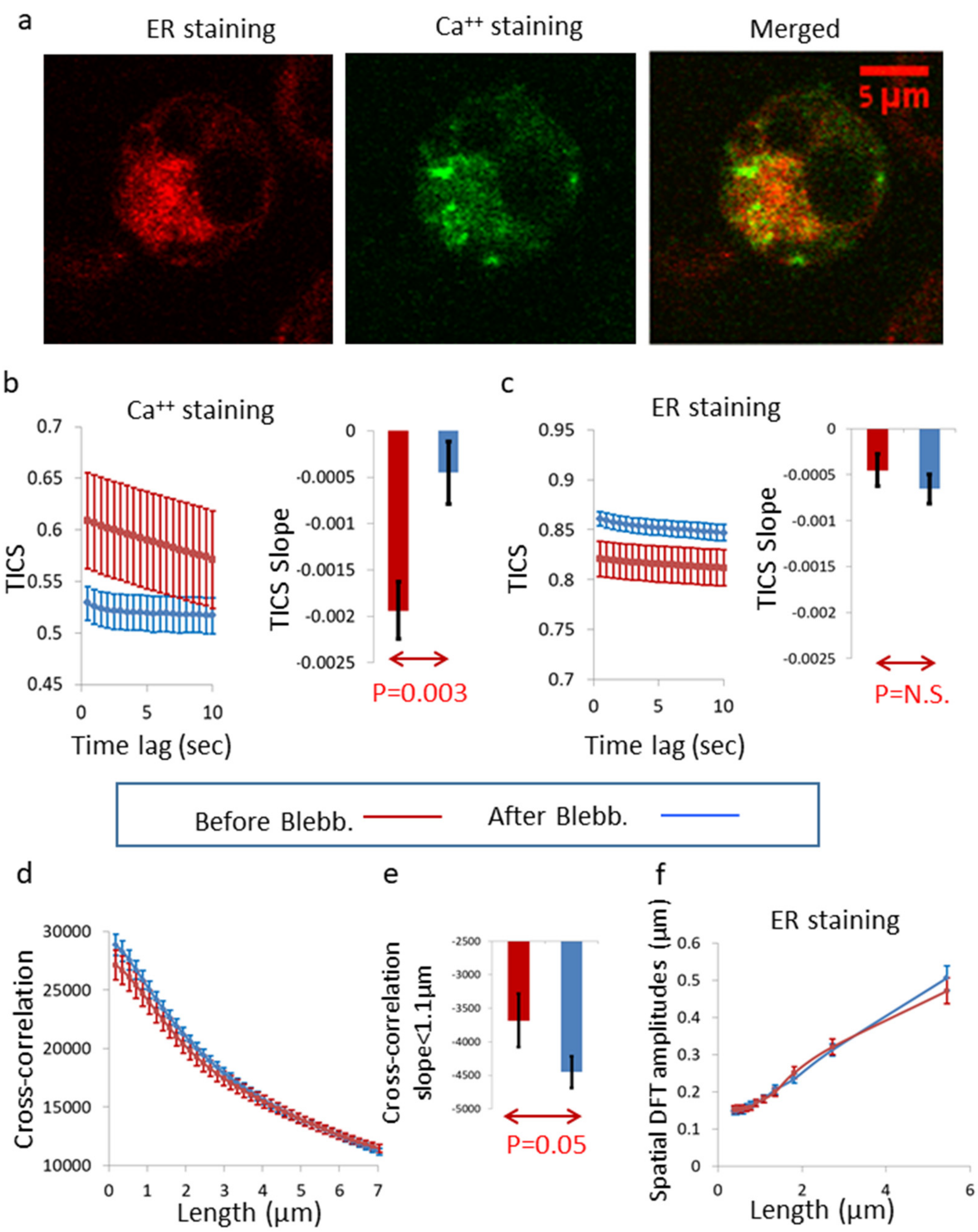

Figure S3. Analysis of spatial and temporal fluctuations of image intensities in live cells (N=20) highlighted with Fluo-4 and ER tracker red utilizing confocal microscopy. (a) ER (using ER tracker red) and Ca^{++} (using Fluo-4) staining in a cell. The average correlation between images of Ca^{++} and ER dyes in each cell using Pearson's coefficient was 0.44 ($P<10^{-4}$). (b) Results of TICS analyses of 100 Ca^{++}-Fluo-4 images with 0.5 sec time interval. On the right, the results of the average TICS slopes in the 20 cells, each cell before and after 10 μM blebbistatin treatment. (c) TICS analysis results of 100 ER tracker red images with 0.5 sec time interval. On the right, the results of the average TICS slopes in the 20 cells, each cell before and after 10 μM blebbistatin treatment. (d) The average spatial cross-correlation results of the two staining channels (Fluo-4 and ER tracker red) in the 20 cells. (e) The average slopes of the decays of the cross-correlation results at length <1.1μm in the 20 cells, before and after blebbistatin. (f) Spatial DFT amplitudes of 20 live cells that were highlighted with ER tracker red before and after 10 μM blebbistatin. Error bars in b-f are SEM and P value are shown in b, c and e.

1.4 The Proposed Mechanism in Which Correlations in Pixels Intensity May Follow Correlations in Motion of Highlighted Substrate in an Organelle

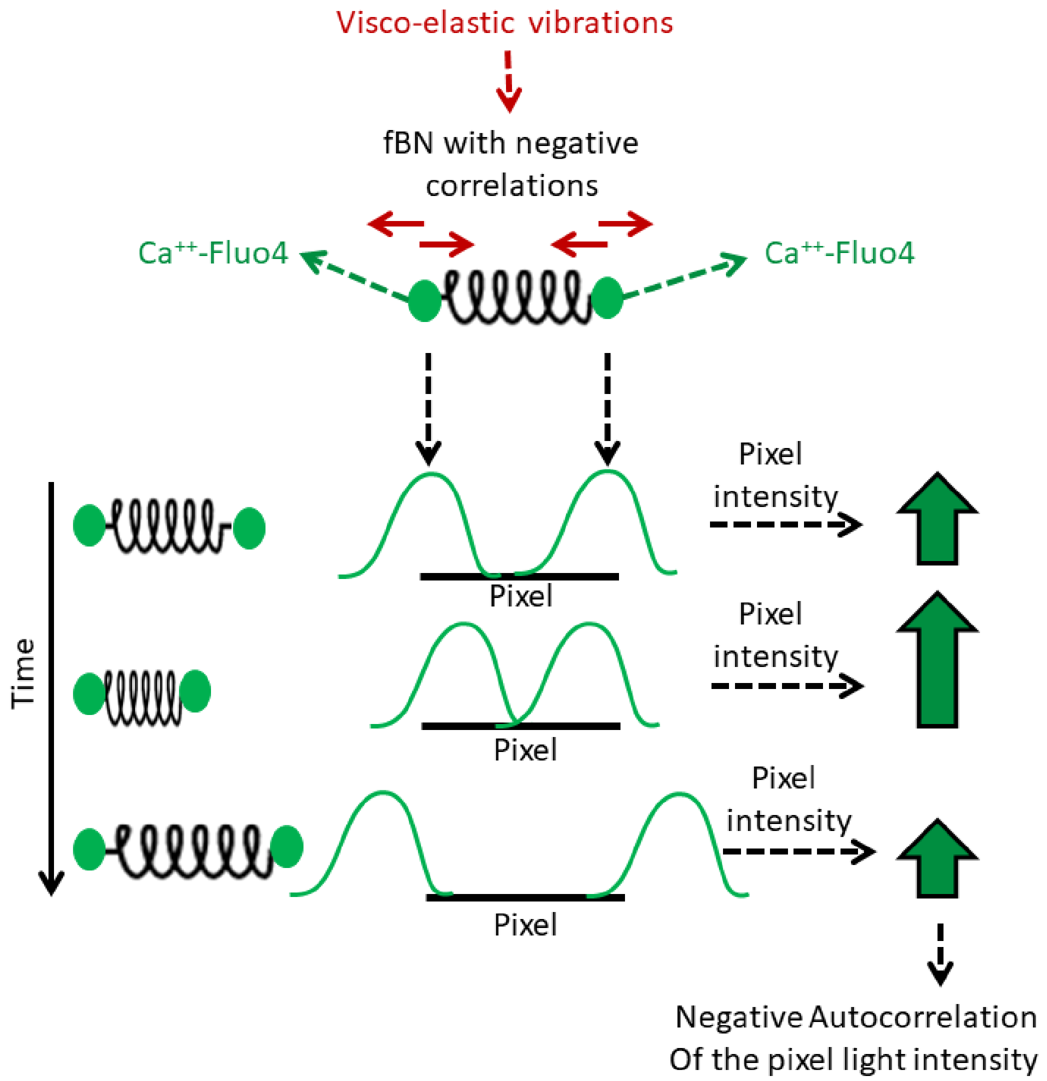

Figure S4. The proposed mechanism for the relation between the viscoelastic vibrating motion of the crowded intra-organelle content, which is highlighted by fluorescence probes, and the autocorrelation values of the temporal fluctuations of pixels light intensity. Under a condition of mechanical work inhibition (via blebbistatin treatment), vibrations in the intra-organelle viscoelastic content will produce negatively correlated vibrating motion of fluorescence probes that are embedded inside it. Each probe is assumed to create an approximated Gaussian of light intensity in the inspecting pixels, while the probes vibrating motion will cause a matched motion of those Gaussians inside the area of the inspecting pixel. Accordingly, the negatively correlated vibrating motion of two nearby probes will produce correspondent negative autocorrelation values in the intensity fluctuations of an inspecting pixel. .

1.5 Simulation of Autocorrelation of fBN in H = 0.3 and H = 0.6 Conditions

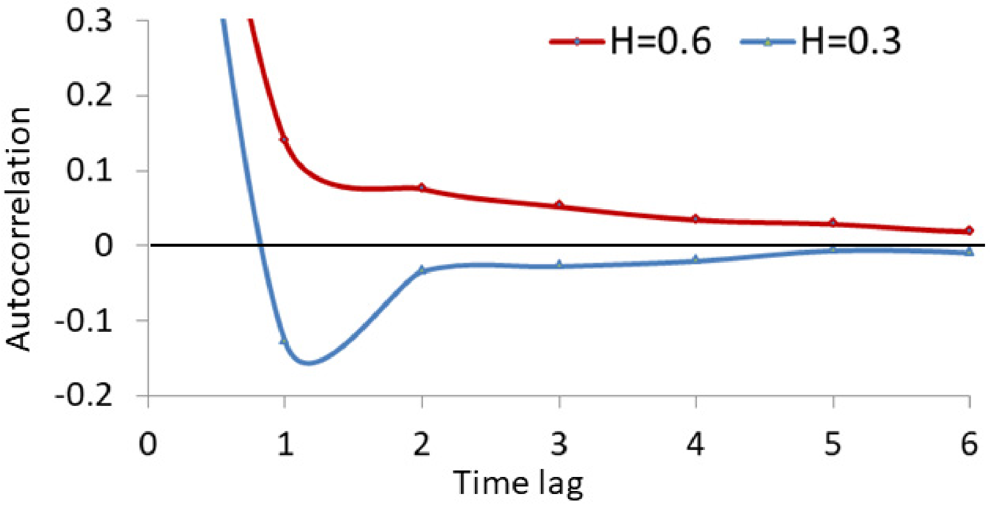

Figure S5. Simulation of autocorrelation values of fractional Brownian Noise (fBN) sequences for two values of H: 0.3 and 0.6. Other parameters for the fBN generation were matched to live cell conditions and were the same as those detailed in section 2.3 of the Results (see also Fig. 5). The number of steps of fBN in each H condition was 10,000. .

1.6 Simulation in Live Cells Conditions of the Probability for a Particle That Conduct fBM in a Finite Interval of 5 Pixels to Be Found in Each Pixel in a Steady State Condition

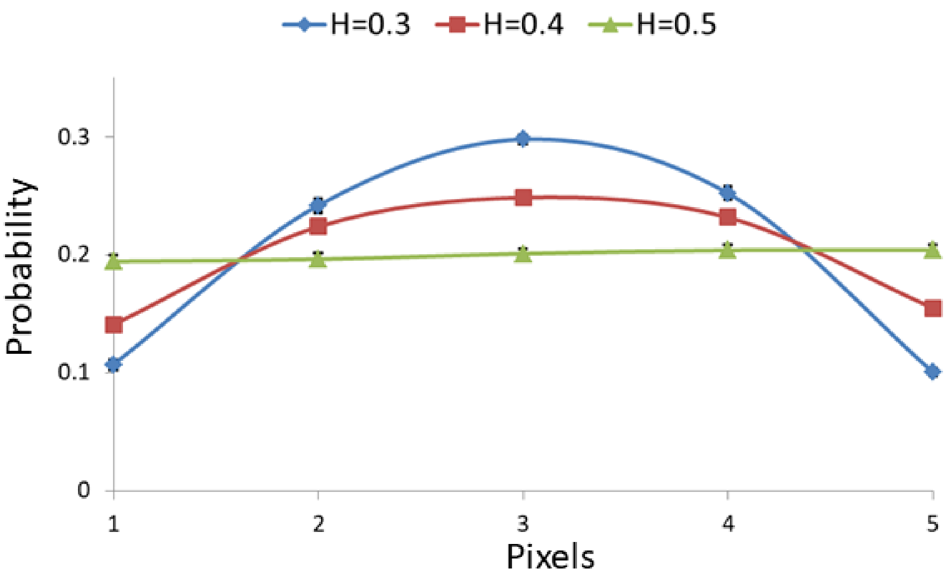

Figure S6. Simulation results of fBM in a finite interval of 5 pixels in a steady state and live cells conditions (as detailed in section 2.3 of the Results). The probability of that particle that conducts fBM to be found in each of the interval pixels is presented and calculated for H values of 0.3, 0.4 and 0.5. The number of steps of fBM for a simulation was 10,000. Ten simulations were conducted for each H value. Error bars are SEM.

1.7 Comparison of the Cells Area before and after Blebbistatin Treatment

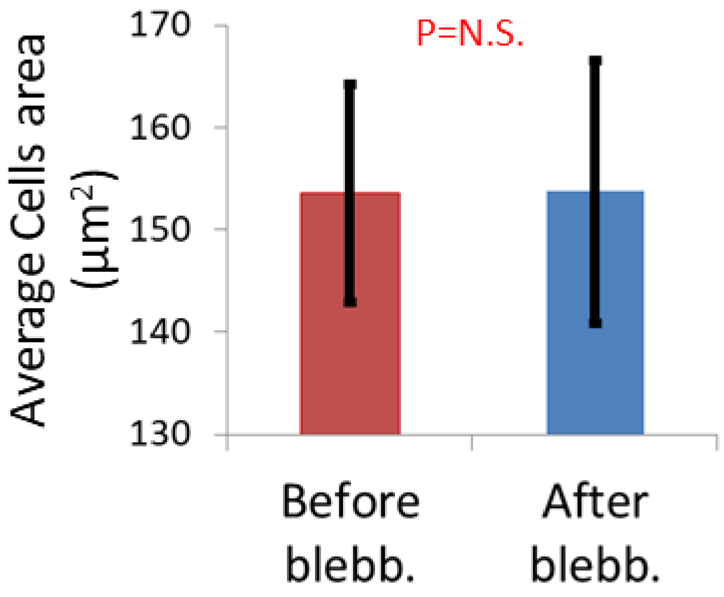

Figure S7. Comparison of the area of the Ca^{++}-Fluo-4-highlighted cells before and after blebbistatin treatment (N=31; as shown in Figure 1). Each cell area was measured before and after 10 μM blebbistatin. Error bars are SEM and P values were not significant (N.S.).
